# Discrepancy in photosynthetic responses of the red alga *Pyropia yezoensis* to dehydration stresses under exposure to desiccation, high salinity, and high mannitol concentration

**DOI:** 10.1007/s42995-021-00115-w

**Published:** 2021-11-25

**Authors:** Guoying Du, Xiaojiao Li, Junhao Wang, Shuai Che, Xuefeng Zhong, Yunxiang Mao

**Affiliations:** 1 Key Laboratory of Marine Genetics and Breeding (Ministry of Education), College of Marine Life Sciences, Ocean University of China, Qingdao, 266003 China; 2 Qingdao West Coast New Area Marine Development Bureau, Qingdao, 266003 China; 3 Key Laboratory of Utilization and Conservation of Tropical Marine Bioresource (Ministry of Education), College of Fisheries and Life Science, Hainan Tropical Ocean University, Sanya, 572022 China

**Keywords:** Dehydration stress, Desiccation, High salinity, High mannitol concentration, Photosynthetic activities, *Pyropia yezoensis*

## Abstract

Macroalgae that inhabit intertidal zones are exposed to the air for several hours during low tide and must endure desiccation and high variations in temperature, light intensity, and salinity. *Pyropia yezoensis* (Rhodophyta, Bangiales), a typical intertidal red macroalga that is commercially cultivated in the northwestern Pacific Ocean, was investigated under different dehydration stresses of desiccation, high salinity, and high mannitol concentration. Using chlorophyll fluorescence imaging, photosynthetic activities of *P*. *yezoensis* thalli were analyzed using six parameters derived from quenching curves and rapid light curves. A distinct discrepancy was revealed in photosynthetic responses to different dehydration stresses. Dehydration caused by exposure to air resulted in rapid decreases in photosynthetic activities, which were always lower than two other stresses at the same water loss (WL) level. High salinity only reduced photosynthesis significantly at its maximum WL of 40% but maintained a relatively stable maximum quantum yield of photosystem II (PSII) (F_v_/F_m_). High mannitol concentration induced maximum WL of 20% for a longer time (60 min) than the other two treatments and caused no adverse influences on the six parameters at different WL except for a significant decrease in non-photochemical quenching (NPQ) at 20% WL. Illustrated by chlorophyll fluorescence images, severe spatial heterogeneities were induced by desiccation with lower values in the upper parts than the middle or basal parts of the thalli. The NPQ and rETR_max_ (maximum relative electron transport rate) demonstrated clear distinctions for evaluating photosynthetic responses, indicating their sensitivity and applicability. The findings of this study indicated that the natural dehydration of exposure to air results in stronger and more heterogeneous effects than those of high salinity or high mannitol concentration.

## Introduction

Macroalgae that inhabit intertidal zones are submerged in and emerged from seawater periodically following the tide cycle. During low tides, macroalgae are exposed to the air for several hours and experience a variety of stressful environmental conditions including desiccation, osmotic stress, high light intensity, high or low temperature, and nutrient limitation (Davison and Pearson 1996). Among these, desiccation has received by far the most attention because of its key function in vertical zonation of macroalgae in coastal areas (Contreras-Porcia et al. 2017 and references in; Davison and Pearson 1996; Dring and Brown 1982; Schonbeck and Norton 1978).

The effects of desiccation on macroalgae have been studied from a wide range of aspects, from basic morphology to physiology, such as rates of survival, growth, photosynthesis, respiration, and reproduction (Contreras-Porcia et al. 2017; Flores-Molina et al. 2014; Karsten 2012; Xu et al. 2019). Using physiological, transcriptomic, and proteomic approaches, several studies have determined that the physiological mechanisms of macroalgae are well coordinated in response to desiccation stress, which includes morphological and cell wall changes, photosynthetic activity diminishment, increased expression and synthesis of associated proteins, scavenging of reactive oxygen species (ROS) by antioxidant enzymes and compounds, and osmolyte synthesis (Contreras-Porcia et al. 2013, 2017; Flores-Molina et al. 2014; López-Cristoffanini et al. 2015). As a central physiological process, photosynthesis is significantly inhibited and decreases immediately under desiccation (Blouin et al. 2011; Dring and Brown 1982; Lipkin et al. 1993). During the water loss process in intertidal macroalgae, photosynthetic pigments and related RNAs and proteins, such as phycobiliproteins, ribulose-1, 5-bisphosphate carboxylase/oxygenase (Rubisco), photosystem I and II proteins, and ferredoxin-NADP^+^, are down-regulated (Contreras-Porcia et al. 2013; Kumar et al. 2011; López-Cristoffanini et al. 2015).

In research studies on dehydration stress, salt and mannitol are commonly used as osmolytes with high solubility that are characteristically transported readily through cell walls and membrane (Chaves et al. 2009; Iwamoto and Shiraiwa 2005). Being able to reduce the cellular water potential, this kind of osmotic dehydration is comparable to desiccation and is commonly utilized to simulate desiccation stress with the benefit of their ease of control and application (Karsten 2012; Munns 2002). However, during natural desiccation by exposure to air, cellular ionic concentrations increase, but the ion ratios remain constant. Furthermore, when using osmolytes, as osmotic stress is prolonged, toxic effects might be induced from excess ions or osmolytes (Bartels and Sunkar 2005; Kirst 1990). On the other hand, most macroalgae could use inorganic ions and small organic osmolytes to create an internal osmotic potential without incurring metabolic damage (Karsten et al. 1997, 1999; Kirst 1990; Yancey 2005). Therefore, it should be considered that in microalgae, different response mechanisms exist to different causes of desiccation.

*Pyropia yezoensis* is a commercially important macroalga that inhabits the intertidal zone and is widely cultivated in countries of East Asia, such as China, Japan, and South Korea (Blouin et al. 2011; Gao et al. 2019; Kim et al. 2017). Like its congeners and members of sibling genera *Porphyra* belonging to the same order Bangiales, *Pyropia* has an extraordinary capacity to withstand the harsh physical and chemical stresses of the upper intertidal zone. Adaptations to these variable environmental conditions include the ability of *P*. *yezoensis* thalli to tolerate water losses of up to 85% and to recover metabolic activities upon rehydration within half an hour (Mao et al. 2019; Terada et al., 2020). In addition, due to the simple, plastic morphology of its gametophyte thalli that comprises a single layer cells of 35–45 µm thick, *P*. *yezoensis* could be the ideal model species for studying the effects of environmental stress.

Using a chlorophyll fluorescence imaging technique, non-invasive monitoring of photosynthetic activities can be realized synchronously for several samples (Baker 2008; Murchie and Lawson 2013). In the present study, using this technique, we aimed to explore dehydration effects on *P. yezoensis* photosynthesis under different treatments of desiccation, high salinity, and high mannitol concentration, and compare photosynthetic responses of *P. yezoensis* to different dehydration stresses. Based on these, further assessment was carried on the feasibility of simulating natural desiccation on macroalgae using osmotic dehydration under high salinity or high mannitol concentration.

## Results

### Photosynthetic capacity based on quenching curves

**Fig. 1:**
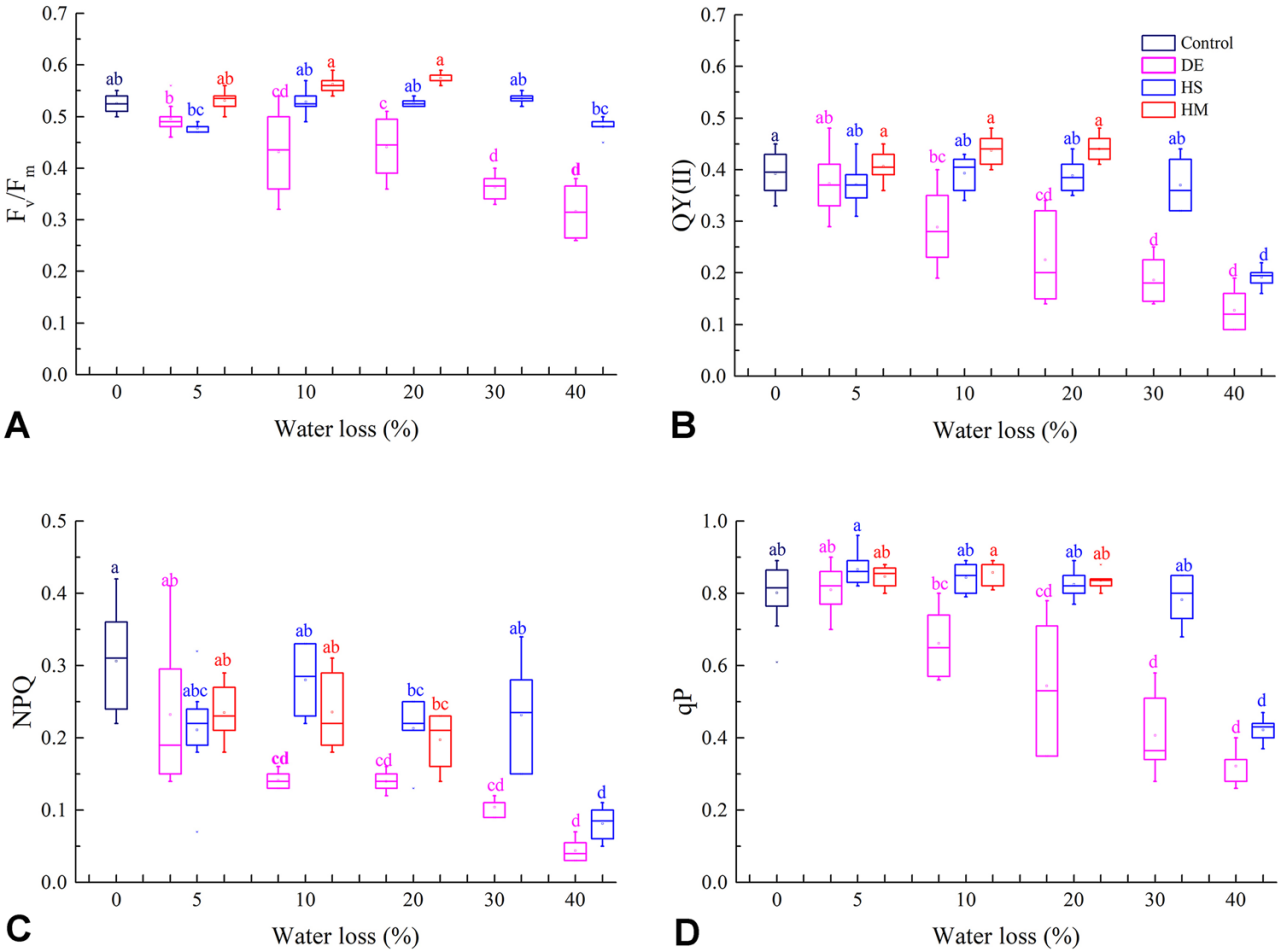
Comparison of photosynthetic parameters of *P. yezoensis* thalli under different dehydration treatments. **A** F_v_/F_m;_ **B** QY(II); **C** NPQ; **D** qP; *DE* desiccation, *HS* high salinity, *HM* high mannitol concentration. Different letters indicate significant difference (*P* < 0.05)

The *P. yezoensis* thalli treated by desiccation presented a rapid decrease in photosynthetic activity along with increased water loss (WL). Except 5% WL, all dehydration levels showed significantly lower F_v_/F_m_ than that of 0% WL (*P* < 0.05). Under 40% WL, the F_v_/F_m_ dropped to 0.45 ± 0.06, which was 13.2% lower than that of 0% WL (Fig. 1A). Even more rapid decreases were recorded based on other photosynthetic parameters, i.e., QY(II), NPQ, and qP. Compared to 0% WL, under 40% WL, the QY(II) was 48.7% lower at 0.20 ± 0.04 (Fig. 1B), NPQ was 87.1% lower at 0.04 ± 0.01 (Fig. 1C) and qP was 59.8% lower at 0.32 ± 0.04 (Fig. 1D).

The effects of dehydration caused by high salinity differed from those caused by desiccation treatment. Dehydration levels from 5 to 40% WL had no significant effect on F_v_/F_m_ (*P* > 0.05) (Fig. 1A). Compared to 0% WL, no significant difference in QY(II) or qP (*P* > 0.05) was observed except at 40% WL (Fig. 1B, D). NPQ decreased but not significantly at 5% WL compared to 0% WL (*P* > 0.05) (Fig. 1C).

In high mannitol concentration treatments, no distinct dehydration effect on F_v_/F_m_, QY(II) or qP of *P. yezoensis* thalli was observed; indeed, there was even a slight increase in activity below 20% WL (*P* > 0.05, Fig. 1A, B, D). Only NPQ exhibited a decrease and was significantly lower at 20% WL compared to 0% WL (*P* < 0.05) (Fig. 1C).

The three kinds of treatments caused different effects on *P. yezoensis* thalli with increased water loss levels (Fig. 1). Comparing at each WL level, the differences were revealed more distinctly. Generally, the thalli treated by desiccation were significantly lower than other two treatments except 5% WL (Fig. 1). Algae exposed to high mannitol concentration always had slightly higher F_v_/F_m_, QY(II) and qP than those exposed to high salinity treatment from 0 to 20% WL (*P* > 0.05) (Fig. 1). Generally, the thalli treated by desiccation had a larger interquartile range (IQR) for F_v_/F_m_, QY(II), and qP than those exposed to the other two treatments. The NPQ of thalli exposed to high salinity showed larger IQR at 10% and 30% WL, and higher median at 10% and 20% WL (*P* > 0.05), than those exposed to high mannitol concentration (Fig. 1C).

### Photosynthetic activity based on rapid light curves (RLCs)

**Fig. 2:**
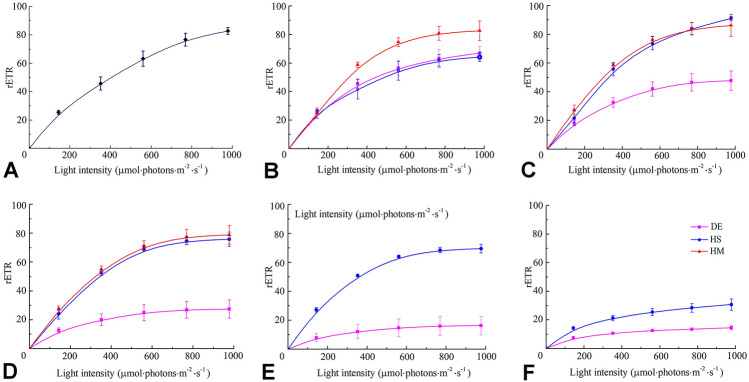
RLCs of *P. yezoensis* thalli with different dehydration treatments at different water loss levels. **A** 0%; **B** 5%; **C** 10%; **D** 20%; **E** 30%; and **F** 40%. *DE* desiccation, *HS* high salinity, *HM* high mannitol concentration

Along with increased dehydration levels, the rapid light curves (RLCs) of *Pyropia* thalli presented large divergences between different treatments (Fig. 2). The rETR of the thalli treated by desiccation decreased more quickly than other two treatments at each WL level. The thalli treated with high salinity did not present significantly lower rETR at WL levels below 40%. The thalli treated with high mannitol concentration resembled the RLCs from 5 to 30% WL.

**Fig. 3:**
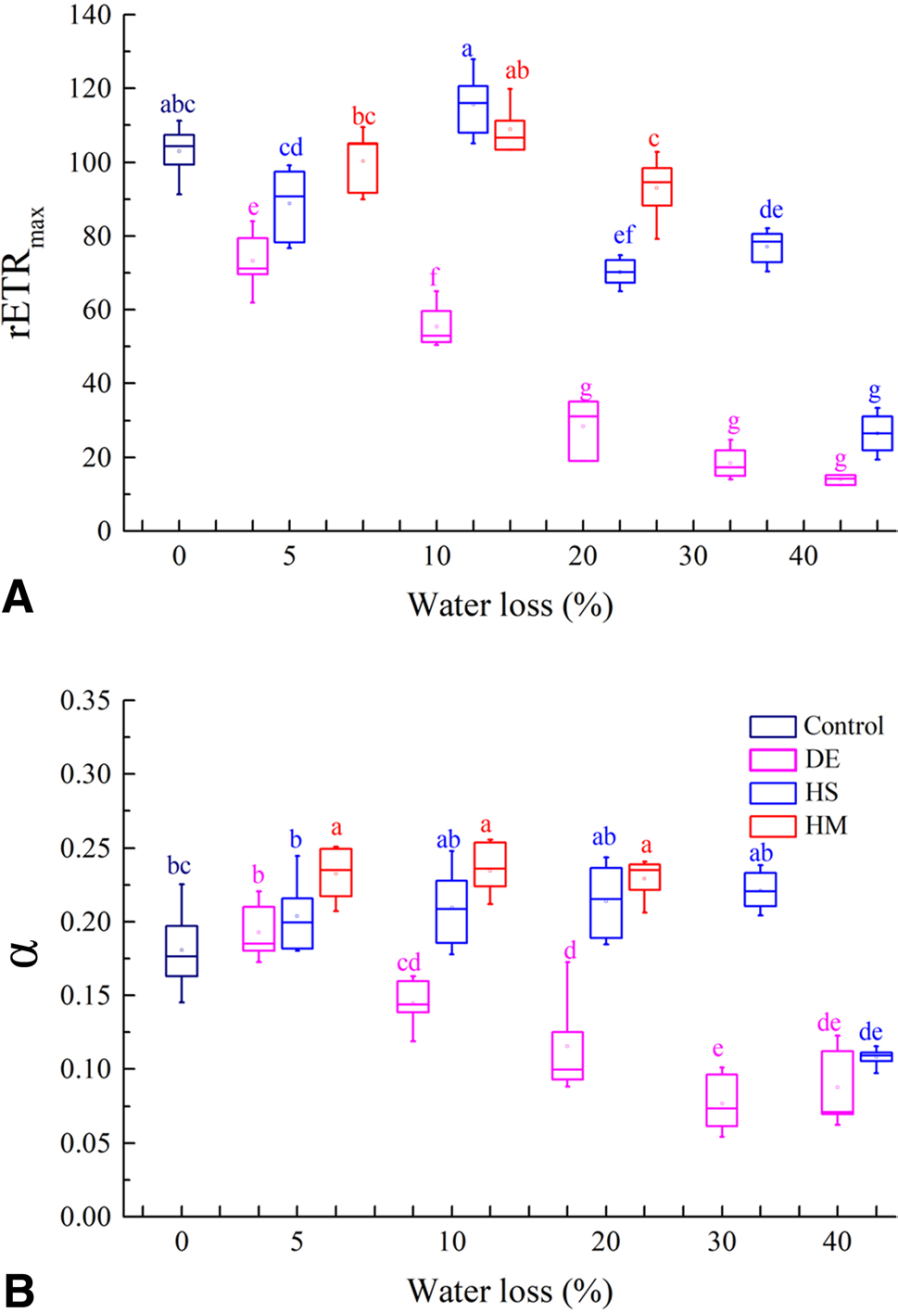
rETR_max_ and α of *P. yezoensis* thalli under different dehydration treatments. *DE* desiccation; *HS* high salinity; *HM* high mannitol concentration. Different letters indicate significant difference (*P* < 0.05)

The rETR_max_ and *α* derived from RLCs varied among the three dehydration treatments (Fig. 3). The rETR_max_ of the thalli treated by desiccation descended with increasing WL, differed significantly from 0% WL (*P* < 0.05), and was always lower than other treatments at the same WL level. Thalli exposed to high salinity had a large variation in rETR_max_ with a significant increase at 10% WL compared to 5% and 20% WL (*P* < 0.05). The rETR_max_ of thalli exposed to high mannitol concentration varied only slightly from 5 to 20% WL (*P* > 0.05). The *α* of desiccated thalli exhibited a decreasing trend similar to the rETR_max_ and also was lower than that of other treatments at each WL level. Thalli exposed to high salinity had a slightly higher *α* although differences from 5 to 30% WL were not significant (*P* > 0.05), whereas the decrease at 40% WL compared to 0% WL was significant (*P* < 0.05). The treatment with high mannitol concentration induced a significant increase in α from 5 to 20% WL compared to 0% WL (*P* < 0.05).

### Spatially heterogeneous effects

**Fig. 4:**
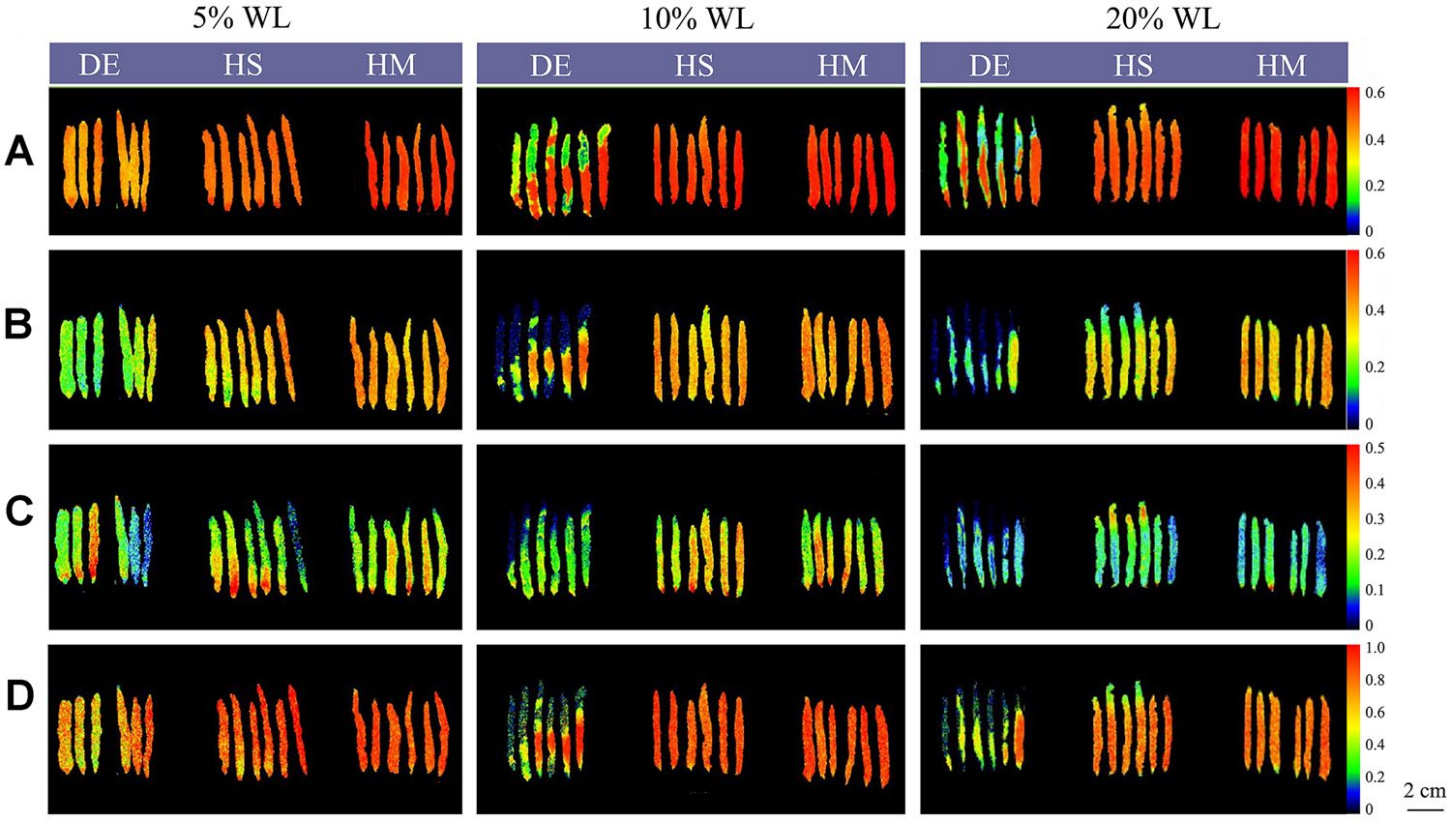
Chlorophyll fluorescence images of F_v_/F_m_ (**A**), QY(II) (**B**), NPQ(**C**), and qP (**D**) at 5%, 10%, and 20% water loss levels under different dehydration treatments (*DE* desiccation, *HS* high salinity, *HM* high mannitol concentration). Within each panel, *P. yezoensis* thalli are oriented with the holdfast at the bottom

Chlorophyll fluorescence images of whole thalli enabled the variations in Fv/Fm, QY(II), NPQ, and qP to be presented visually (Fig. 4). Although showing the same trends as those presented in Fig. 1, the spatial heterogeneity of individual parameters displayed discrepancies among thalli under different treatments at 5%, 10%, and 20% WL. The thalli exposed to high salinity and high mannitol concentration showed relatively homogenous values, with slight differences at the very basal part of the holdfast and at the tip of the thallus. However, thalli exposed to air showed large heterogeneity for all four parameters with much lower values in the upper part, especially at 10% and 20% WL. Furthermore, for any given treatment, differences among individual thalli were larger when treated by desiccation than the other two treatments, resulting in larger standard deviations of parameters, as shown in Fig. 1.

## Discussion

Red macroalgae of the genus *Porphyra* and *Pyropia* are considered as marine crops shaped by stress (Blouin et al. 2011). Many species of *Porphyra* and *Pyropia* can lose 85–95% of their cellular water during the daytime low tide in intertidal zones when exposed to air for several hours. During the exposure time, desiccation along with other stressors such as high irradiance and salinity inhibits photosynthesis of *Porphyra* or *Pyropia* thalli and this effect increases with increased water loss (Blouin et al. 2011; Lipkin et al. 1993; Terada et al. 2020).

In the present study, thalli dehydrated by desiccation showed significant decreases in their maximum photosynthetic capacity (indicated by F_v_/F_m_). This is consistent with the response of *Pyropia haitanensis* (previously *Porphyra haitanensis*), and of other macroalgae, to desiccation (Flores-Molina et al. 2014; Gao et al. 2013; Xu et al. 2016). Moreover, dehydration can inhibit electron transfer and energy conversion in photosynthetic reaction centers, leading to reductions in other measures of photosynthetic activity such as QY(II) and ETR (Jiang and Gao 2009; Yu et al. 2018). Nevertheless, although there is a general decrease in photosynthetic activity with increasing water loss, subtle discrepancies exist in QY(II), ETR, or NPQ among studies on *Pyropia* species. For instance, Gao et al. (2013) reported that *P. haitanensis* showed increased ETR in both PSII and PSI reaction centers under moderately desiccated conditions (water loss less than 30%), and its NPQ increased significantly at 20–25% water loss. In contrast, Xu et al. (2016) reported that QY(II) and F_v_/F_m_ continuously decreased with increasing water loss in *P. haitanensis*. Slight but not significant increases in QY(II) and ETR in PSII were observed when *P. yezoensis* was exposed to air and directly utilized atmospheric CO_2_ (Zhou et al. 2014). In the present study, there was a general decrease in six parameters and RLCs in thalli under desiccation, with slight but not significant fluctuations. These discrepancies might due to differences in experimental conditions, the characteristics and growth status of testing organisms, or the response mechanism of photosynthetic physiology itself. Nevertheless, a rapid decrease in photosynthesis invariably occurred in *Pyropia* thalli as a result of desiccation.

High salinity stress on *Pyropia* is directly imposed on the whole thallus, unlike in higher plants with well-developed transportation systems connecting their roots, stems and leaves. The water loss under high salinity can occur within a few minutes and is followed by osmotic and ionic stress (Chaves et al. 2009). In the present study, the high salinity treatment, i.e., 100 PSU NaCl-rich seawater, was limited to an exposure time of 10 min which resulted in a maximum individual water loss of 40%. Furthermore, only at 40% WL did the thalli present a significant decrease in most photosynthetic parameters with the except on F_v_/F_m_. The relative stability of F_v_/F_m_ up to 40% WL indicated that the potential photosynthetic capacity of PSII had not yet been interrupted, although effective photosynthetic efficiency and activity were inhibited at 40% WL as indicated by other parameters. Usually, studies on the effects of high salinity on macroalgae use treatment times of hours or even days (Huan et al. 2014; Nitschke et al. 2014; Yu et al. 2018; Zheng et al. 2019). During such time periods, higher osmotic and ionic stresses such as inhibition on enzyme activities due to excess Na^+^ ions would likely affect the thalli (Bartels and Sunkar 2005; Kolomeichuk et al. 2020; Sudhir and Murthy 2004). It has also been reported that when exposed to high salinity for 2 h, *P. yezoensis* can upregulate related enzymes to exploit NAD(P)H as an endogenous electron donor for reducing the plastoquinone pool and stabilizing the ETR (Yu et al. 2018).

In the present study, high mannitol concentration had almost no influence on photosynthesis of *P. yezoensis* thalli, with the exception of a significantly lower NPQ at 20% WL and a significantly higher α than the control. Mannitol is a universal sugar alcohol compound that is present in many photosynthetic organisms, although its biosynthesis is unusual for red algae, since they usually synthesize heteroside, floridoside, isofloridoside (Bangiales), or digeneaside (Ceramiales) (Iwamoto and Shiraiwa 2005; Kremer and Kirst 1982; Kumar et al. 2011). However, the D-mannitol-1-phosphate dehydrogenase (M1PDH) biosynthesis pathways of mannitol were found in red algae of the genera *Caloglossa* (Iwamoto et al. 2003; Karsten and West 1993; Karsten et al. 1997), *Dixoniella* and *Rhodella* (Karsten et al. 1999) and of the family Bangiophyceae (Tonon et al. 2017). In genome analysis carried out in our laboratory (unpublished data), the D-Mannitol dehydrogenase pathway from D-fructose to D-Mannitol (Richter and Kirst 1987), but not the M1PDH pathways, was found to be possibly responsible for mannitol biosynthesis. With mannitol metabolism, the red algae *Caloglossa* could cope with dehydration stress caused by high salinity (Karsten and West 1993; Karsten et al. 1997). Therefore, in the present study, endogenous mannitol might have served as an osmotic pressure regulator by balancing exogenous overdose, which resulted in few effects on photosynthesis and even increased the light utilization efficiency (*α*) of *P. yezoensis* thalli exposed to high mannitol concentrations.

Comparing the three dehydration treatments revealed the presence of distinct responses of photosynthesis in *P. yezoensis* thalli. Adverse effects were more severe and faster in thalli exposed to desiccation than those exposed to high salinity or high mannitol concentration. Furthermore, using chlorophyll fluorescence imaging, it was revealed that thalli treated with desiccation had a higher spatial heterogeneity in photosynthesis than other two treatments. In fact, desiccation and osmotic stress due to high salinity or high mannitol concentration reflect two different forms of water deprivation. Under salt or mannitol stress conditions, seaweed cells are still in full contact with liquid water, whereas desiccation due to exposure to air leads to strong cellular dehydration (Karsten 2012). The distinct spatial heterogeneity suggests that more attention should be paid to physiological, biochemical, and gene expression responses to environmental stress. For instance, different parts of the thallus should be considered, especially the tip area which expressed the most adverse effects to desiccation. Although high salinity can be easily applied as substitute for dehydration treatment due to its relatively homogenous effects, it should be kept in mind that the response mechanisms of *P. yezoensis* thalli to direct desiccation and osmotic dehydration are different. Even at the same levels of osmotic stress, markedly different effects were induced by high salinity and high mannitol concentration. Therefore, for macroalgae such as *P. yezoensis*, high salinity or high mannitol concentration could not be used to simulate natural dehydration. Detailed comparisons should be done in advance, as in this and other studies (Iwamoto and Shiraiwa 2005; Slama et al. 2007).

The chlorophyll fluorescence techniques used in the present study provide comprehensive information in a non-invasive and instant way for assessing photosynthetic performance in the thalli of red algae. Quenching curves and RLCs can yield information on F_v_/F_m_, QY(II), NPQ, and ETR in response to various environmental stresses, either on their own or in combination (Adams et al. 1999; Bilger et al. 1995; Maxwell and Johnson 2000). The changes in these parameters are widely used as reliable diagnostic indicators of stress on photosynthesis, especially the F_v_/F_m_, which is the most important parameter for reflecting the potential photosynthetic capacity of PSII (He et al. 1996; Valladares and Pearcy 1997). In the present study, four parameters derived from quenching curves and two parameters from RLCs were used for assessing the effects of dehydration on photosynthesis in the red alga *P. yezoensis*. Among these parameters, NPQ and rETR_max_ exhibited higher variation at different WL levels than the others. This implies that NPQ and rETR_max_ could be as sensitive indicators of photosynthetic performance for assessing stress on macroalgae such as *P. yezoensis*.

## Materials and methods

### Culture of *Pyropia* thalli

The *Pyropia yezoensis* strain RZ was maintained by the Laboratory of Algae Genetics and Breeding (Ocean University of China). *Pyropia yezoensis* thalli were cultured in 2 L bottles with Provasoli’s enriched seawater (PES) medium at 10 °C, under 60 μmol·photons·m^−2^·s^−1^ with a 12:12 light:dark (L:D) cycle. The culture was bubbled with filter-sterilized air constantly and the medium was renewed every three days.

### Dehydration treatment

When the thalli reached 5–7 cm in length, they were randomly selected for three different dehydration treatments, i.e., desiccation, high salinity, and high mannitol concentration. The temperature and light intensity during the dehydration treatments were the same as the culture conditions. Air humidity remained 60%. Excess water on the thalli surface was removed with tissue paper before treatment.

In the treatment of desiccation, the thalli were spread flat on a Petri dish exposing them to air in a cabinet with stable temperature and moisture. For each sample group, the treatment time was controlled to reach water loss of 5%, 10%, 20%, 30%, and 40%, respectively.

In the salt treatment, the thalli were immersed in one of five NaCl solutions (35, 40, 60, 80, and 100) for 10 min to reach the water loss of 5%, 10%, 20%, 30%, and 40%, respectively. A preliminary experiment showed that salinity more than 100 induced only minimally higher water loss and made *P. yezoensis* thalli obviously deformed.

In the high mannitol concentration treatment, thalli were incubated in 0.8 mol/L mannitol seawater for 10 min, 20 min, or 60 min to reach water losses of 5%, 10%, and 20%, respectively. No higher water loss could be obtained using the maximum dissolved concentration of mannitol (0.8 mol/L). Six thalli of each treatment level were used for measuring photosynthetic activity.

### Measurement of water loss

Dehydration levels of thalli were determined using the formula: ${\text{Water}}\;{\text{loss}}\;\left( {{\text{WL}}} \right) = \;\left( {W_{0}  - \;W_{t} } \right)/\left( {W_{0}  - \;W_{d} } \right)\; \times \;100\%$, where *W*_0_ is the initial weight after the removal of excess water, *W*_*t*_ is the weight of thalli at time t after dehydration, and *W*_*d*_ is the dry weight of thalli after drying to constant weight at 60 °C (Kim et al. 2009).

### Determination of photosynthetic activity

Photosynthetic activity of thalli was measured using a chlorophyll fluorescence imaging system (FluorCAM MF800, PSI, Czech). The samples were dark-adapted for 10 min before measurements were taken. For quenching curve analysis, the saturating pulse was set as 45% (1892.65 μmol·photons·m^−2^·s^−1^), and actinic light was set as 23% (58.24 μmol·photons·m^−2^·s^−1^). Parameters of F_v_/F_m_ (maximum PSII quantum yield), QY(II) (effective PSII quantum yield), NPQ (non-photochemical quenching), and qP (photochemical quenching) were derived from export data of quenching analysis in operating software. The rapid light curves (RLCs) were measured by exposure to six incremental steps of actinic light ranging from 0 to 980 µmol photons m^−2^ s^−1^ (PAR, photosynthetically active radiation). For each irradiance level (*E*), the relative electron transport rates (rETR) were calculated from the product of *E* and the PSII effective quantum yield (Y(II)), rETR = *E* × Y(II) (Genty et al. 1989). RLCs were fitted to the model from (Harrison and Platt (1986) and derived corresponding parameters, rETR = rETR_max_ [1-exp (− *α* × *E*/rETR_max_)], where rETR_max_ is the maximum rETR, α is the maximum light utilization coefficient, and *E* is the light intensity.

### Statistical analysis

All the data were expressed as a mean value of six replicates with standard deviation (SD). One-way ANOVA and Tukey tests were used to analyze the differences among treatments using SPSS 17.0 (SPSS Statistics 17.0, IBM). The significance level was set at 0.05.
